# Effects of delaying binge drinking on adolescent brain development: a longitudinal neuroimaging study

**DOI:** 10.1186/s12888-016-1148-3

**Published:** 2016-12-13

**Authors:** Josiane Bourque, Travis E. Baker, Alain Dagher, Alan C. Evans, Hugh Garavan, Marco Leyton, Jean R. Séguin, Robert Pihl, Patricia J. Conrod

**Affiliations:** 1 Centre de recherche CHU Sainte-Justine, 3175 Côte Ste-Catherine, Montreal, Québec H3T 1C5 Canada; 2 Department of Psychiatry, Faculty of Medicine, University of Montreal, Montreal, Québec Canada; 3 Center for Molecular and Behavioural Neuroscience, Rutgers University, Newark, NJ USA; 4 Montreal Neurological Institute, McGill University, Montreal, Québec Canada; 5 Department of Psychiatry, University of Vermont, Burlington, VT USA; 6 Department of Psychiatry, McGill University, Montréal, Québec Canada; 7 Department of Psychology, McGill University, Montréal, Canada

**Keywords:** Neurodevelopment, Alcohol use, Adolescence, Preventative intervention program, Functional imaging, Structural imaging

## Abstract

**Background:**

Onset of alcohol use by 14 relative to 21 years of age strongly predicts elevated risk for severe alcohol use problems, with 27% versus 4% of individuals exhibiting alcohol dependence within 10 years of onset. What remains unclear is whether this early alcohol use (i) is a marker for later problems, reflected as a pre-existing developmental predisposition, (ii) causes global neural atrophy or (iii) specifically disturbs neuro-maturational processes implicated in addiction, such as executive functions or reward processing. Since our group has demonstrated that a novel intervention program targeting personality traits associated with adolescent alcohol use can prevent the uptake of drinking and binge drinking by 40 to 60%, a crucial question is whether prevention of early onset alcohol misuse will protect adolescent neurodevelopment and which domains of neurodevelopment can be protected.

**Methods:**

A subsample of 120 youth at high risk for substance misuse and 30 low-risk youth will be recruited from the Co-Venture trial (Montreal, Canada) to take part in this 5-year follow-up neuroimaging study. The Co-Venture trial is a community-based cluster-randomised trial evaluating the effectiveness of school-based personality-targeted interventions on substance use and cognitive outcomes involving approximately 3800 Grade 7 youths. Half of the 120 high-risk participants will have received the preventative intervention program. Cognitive tasks and structural and functional neuroimaging scans will be conducted at baseline, and at 24- and 48-month follow-up. Two functional paradigms will be used: the Stop-Signal Task to measure motor inhibitory control and a modified version of the Monetary Incentive Delay Task to evaluate reward processing.

**Discussion:**

The expected results should help identify biological vulnerability factors, and quantify the consequences of early alcohol abuse as well as the benefits of early intervention using brain metrics.

## Background

Harmful alcohol use begins in adolescence, a period characterized by developments in cognition, behavior and brain maturation. Onset of alcohol use by 14 relative to 21 years of age strongly predicts elevated risk for severe alcohol use problems, with 27% versus 4% of individuals exhibiting alcohol dependence within 10 years of onset [1]. The reasons for this increased vulnerability to alcohol effects in youth need to be clarified.

Important maturational changes in brain anatomy, connectivity, and function continue well into late adolescence. Longitudinal empirical evidence suggests that brain maturation in both grey and white matter follows a postero-anterior trajectory across the cortex [2–4], where frontal areas, and white matter tracts that originate there, mature in parallel with higher order executive functional changes in later adolescence (e.g., inhibitory performance [5] or working memory [6]), relative to other, more basic cognitive functions (e.g., attention). On a functional basis, there is preliminary evidence for the dual system model [7, 8], in which top-down cognitive control networks develop linearly with age between childhood and adulthood [9, 10], while the reward sensitivity system (striatum, medial and orbital prefrontal cortices) follows a non-linear maturation that eventually caps by mid-adolescence [11].

When looking at the effects of alcohol misuse on brain maturation, neuropsychological studies in youth suggest that early alcohol abuse correlates with cognitive impairments in verbal, non-verbal and spatial working memory tasks, as well as attention and executive functions [12–14]. At the brain level, an association was found between smaller hippocampal volumes and early onset of alcohol dependence in adolescence, possibly accounting for the memory deficits noted above [15]. Moreover, functional neuroimaging studies have detected abnormal brain activations in adolescents with an alcohol use disorder, relative to healthy volunteers, when they were tested during spatial working memory tasks even though task performance did not differ [16, 17]. These findings suggest that the combination of neuroimaging with cognitive measures might be needed to capture the subtle developmental effects of adolescent onset alcohol misuse, which might translate to future addiction.

### Neurodevelopmental profiles of at-risk youth for alcoholism

#### Impulsivity (IMP)

Two major risk factors for adolescent onset of alcohol misuse are a family history of alcoholism and disinhibited psychopathology, both of which are characterized by a failure of self-control [18]. Various higher order cognitive functions that also involve inhibitory control, such as working memory and delay discounting, as well as their mediating fronto-striatal (inhibition), fronto-parietal (working memory) and striato-limbic (reward sensitivity) neural networks, are abnormal in children with disinhibited personalities and childhood disorders of impulsiveness [19–23]. Our team recently reported that IMP in 14-year olds is inversely associated with grey matter volume in the orbitofrontal cortex [24] and reduced activity within bilateral frontal brain regions during failed inhibition [25]. Furthermore, IMP and brain-related measures during failures of inhibition were prospectively predictive of substance misuse and disinhibited psychopathology over a two-year period in adolescence. Finally, there is some preliminary evidence for an interaction effect, suggesting that individuals prone to alcohol dependence, due to family history of alcoholism, are particularly susceptible to the effects of alcohol on global cognitive function [26].

#### Sensation seeking (SS)

The desire for intense and novel experiences, or SS [27], is associated with risk-taking and reckless behavior among youth [28], with heavier drinking [29, 30] and with risk for adverse drinking consequences [31]. We recently reported that SS is associated with a pattern of responding to cognitive/motivational tasks that involves a sensitivity to reward that is predictive of binge drinking over and above the contribution of co-occurring conduct problems [32]. SS and IMP appear to differentiate adolescent substance use behavior: SS is associated with early onset use, binge drinking and drug experimentation, but IMP is associated with higher quantity of use after substance use onset [33]. At the neurobiological level, we also showed that these clinical profiles could be dissociated using brain activation patterns during failed response inhibition (IMP) vs. response to reward anticipation (SS) [25]. Existing neuroimaging studies with high-risk adolescents are, for the most part, cross-sectional, so it remains unclear whether the structural and functional deficits attributed to the effects of alcohol misuse exist in individuals at risk for alcoholism prior to the onset of alcohol misuse or if early onset use causes the development of new deficits over and above existing neurocognitive abnormalities, or in interaction with such abnormalities.

### The Preventure Programme

The Preventure Programme is a school-based alcohol prevention programme targeting four specific personality risk factors for adolescent alcohol misuse: impulsivity, sensation seeking, hopelessness and anxiety sensitivity [34, 35]. The Substance Use Risk Profile Scale (SURPS) [36] has been developed by our team to enable us to identify high-risk youth based on these personality risk dimensions [29, 37]. The intervention programme, based on a cognitive-behavioral model, involves delivering two 90-min group sessions to adolescents who screen positive on one of these four personality traits in school screening. Five randomized controlled trials with high school students revealed that personality-targeted interventions reduce rates of drinking and binge drinking by up to 50% [34, 38, 39], and these effects have been shown to last up to 3 years [35, 40, 41]. Despite these very promising findings, these outcomes may underestimate the true benefits of early intervention as our previous evaluations did not assess brain function and maturation.

## Methods

### Objectives and hypotheses

In 2011, the Co-Venture Trial was funded by the Canadian Institutes of Health Research (CIHR) to test the 4-year impact of the Preventure programme on delaying early onset drinking and binge drinking in students as well as long-term addiction outcomes. A subsequent CIHR grant was awarded for the Neuroventure study, allowing us to add longitudinal neuroimaging measures to test the following objectives/hypotheses:

1. Compare high-risk (IMP and SS) and low-risk youth on measures of brain structure and function to quantify neurocognitive endophenotypes of these two risk profiles prior to exposure to alcohol in adolescence. We expect to observe impaired fronto-striatal connectivity and function during response inhibition in IMP as well as altered function of frontal and sub-cortical regions (i.e., basal ganglia and ventral striatum) during reward anticipation in SS.
2. Measure the correlates of adolescent drinking and binge drinking on the development of brain structure and function in adolescence, controlling for baseline differences in personality, cognitive profiles, and neural measures that are implicated in the predisposition to early onset alcohol misuse. By comparing youth who take up drinking to those who do not (regardless of intervention status), we will test the extent to which binge drinking trajectories are associated with delayed or altered development of fronto-striatal and fronto-limbic networks that mediate response inhibition and anticipation of reward/punishment. Three hypotheses will be examined. i) Global effects: abnormalities will be observed systematically across all measures of brain function and structure, ii) Developmental specificity: alcohol-induced abnormalities will be specific to regions and cognitive processes that are maturing in adolescence, and iii) Interaction effect: alcohol will exacerbate the abnormalities shown to characterize each high-risk group at baseline.
3. Test causal hypotheses by testing whether delaying drinking (through the Preventure Programme) can produce observable protective effects on structural and functional brain development. By comparing high-risk youth who received intervention to those who did not, we will estimate an intervention effect on structural and functional brain outcomes, which again might be expressed globally across the brain, or more specifically in those areas developing during adolescence or most abnormal in high-risk groups (as revealed in objective 1).

### The Co-Venture Trial (from which the participants are recruited)

This study uses a cluster-randomized controlled design in which 31 schools (cluster) agreed to conduct annual surveys for 5 years with all consenting adolescents (*N* = 3826) who were enrolled in Grade 7 in September 2012. Schools were randomly assigned to i) be trained and assisted in the delivery of the Preventure programme to Grade 7 high-risk adolescents or ii) not be exposed to the intervention. Adolescents were screened using the SURPS [36] during their Grade 7 year and invited to participate in personality-specific Preventure workshops if they scored one standard deviation above the school mean on one of the four SURPS dimensions. The Co-Venture clinical Trial is registered on www.ClinicalTrials.gov, “Co-Venture: a Cluster Randomized Trial Investigating the Effects of Selective Intervention on Adolescent Cognitive Development and Addiction”, study NCT01655615.

### Neuroventure: neuroimaging add-on study

#### Participants

**Fig. 1:**
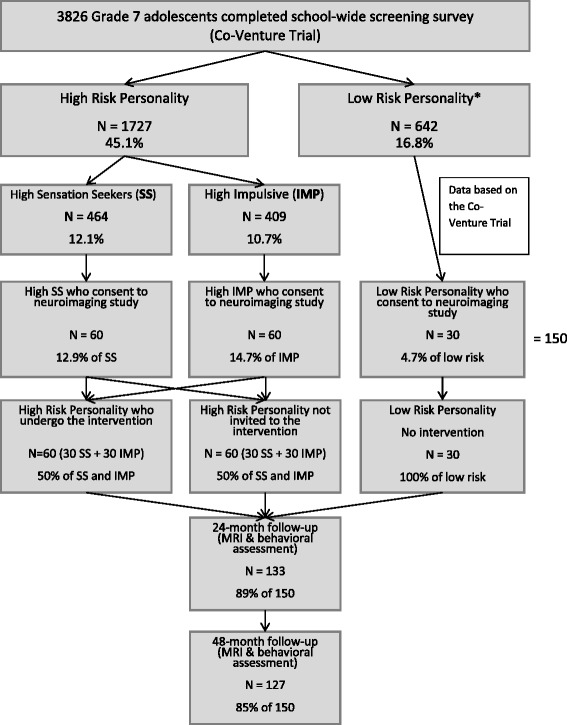
Consort diagram for the Neuroventure Study. *For the Neuroventure project, we considered a low-risk personality to have a z-score below 0.5 on both Negative thinking (NT) and Anxiety sensitivity (AS) traits, and a z-score below 0.0 on both SS and IMP traits, to be adequate matches for the SS and IMP groups

**Fig. 2:**
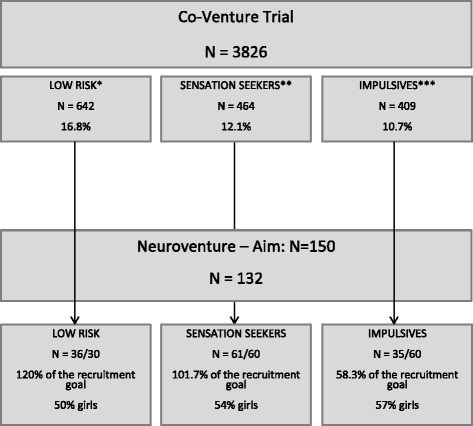
Recruitment data in the still ongoing project. *For the Neuroventure project, we considered a low-risk personality to have a z-score below 0.5 on both NT and AS traits, and a z-score below 0.0 on both SS and IMP traits, to be adequate matches for the SS and IMP groups. **A z-score above 1.0 on the SS personality trait. ***A z-score above 1.0 on the IMP personality trait

Neuroventure will focus on high-risk students with SS and IMP personality profiles as they contribute most to the prediction of early onset substance-related behaviors in adolescence [42]. Thus, a subsample from the Co-Venture Trial of 60 high SS (30 of whom will receive the intervention), 60 high IMP (30 of whom will receive the intervention) and 30 low-risk (low SS and low IMP will not be invited to participate in the interventions) Grade 7 students (50% girls) will be invited to participate in this neuroimaging study (Please refer to Fig. 1 for the consort diagram and to Fig. 2 for the actual recruitment data). An independent informed consent from parents and adolescents will be obtained for the present study. Exclusion criteria will include major neuro-developmental disorders (e.g., autism), uncorrectable visual impairment or hearing deficits, severe mental health problems (e.g., schizophrenia, bipolar disorder), uninterruptable central nervous system medication, and any magnetic resonance imaging (MRI) contraindications (e.g., pregnancy, braces, etc.).

#### Procedure

Eligible students will be invited to attend three identical scanning sessions over a 5-year period: the first session during the baseline year of the Co-Venture Trial (for intervention schools, this would refer to the period prior to when intervention sessions are run), and second and third session 24 and 48 months later, respectively (Grade 9 and 11). Participants will undergo a 65-min structural and functional neuroimaging assessment, and will complete a 2-h battery of neuropsychological tasks post-scan. In addition, participants will be assessed on substance use, global IQ and behavioral measures annually for 4 years post intervention through the Co-Venture Trial.

#### Behavioral assessment

Adolescents will be administered the Timeline Follow-Back Interview [43, 44] to assess past 6 months alcohol and drug use in a face-to-face interview.

To evaluate cognitive functioning, adolescents will complete the STROOP [45], a measure of response inhibition. To evaluate non-spatial and spatial working memory, participants will be administered the Self-Ordered Pointing Test (SOPT; [46]), and the N-back task [47] (the 2- and 3-Back versions). The Children’s memory scale (CMS)-Dot Location [48], a sub-test of the CMS, will be used to assess spatial immediate and deferred memory recall. The participants will also complete the computerized Balloon Analogue Risk Task (BART: [49]). The BART assesses real-world risk taking behavior by balancing the potential for reward (inflating the balloon) versus loss (balloon exploded). Finally the sub-tests of the Vocabulary and Block Design will be used as an estimate of the Wechsler Intelligence Scale for Children 4th Edition [50] full scale IQ.

#### Scanning protocol

Functional MRI (fMRI) data will be acquired on a 3T Siemens Magnetom Trio MRI scanner using whole brain gradient echo planar imaging (EPI) sequence sensitive to blood oxygenation level dependent (BOLD) contrast in 47 slices (Repetition time (TR) = 2.5 s; Echo time (TE) = 30 ms; 3 mm thickness; voxel size: 3 × 3 × 3 mm^3^). A higher resolution structural scan using an ultrafast gradient echo 3D sequence (MPRAGE) will also be collected for structural analyses and to aid functional data registration (TR = 2.3 s; TE = 2.96 ms; 1 mm thickness; voxel size: 1 × 1 × 1 mm^3^). Diffusion weighted imaging (DWI) data will be acquired during a peripherally gated spin echo EPI sequence with diffusion sensitization gradients (‘b value’ of 100 s/mm^-2^) applied in 64 directions (HARDI protocol); this will provide whole brain coverage at 2.0 mm isotropic resolution.

Rapid, event related, BOLD fMRI will be performed while participants complete tasks that engage two specific domains of cognitive performance: a modified version of the Monetary Incentive Delay Task (MIDT) and a motor response inhibition task (Stop-Signal task) (for further details please refer to [51]). The MIDT has been used extensively to investigate changes in neural activity in response to different stages of reward processing (e.g., anticipation, outcome processing, and consumption), as well as the processing of tasks under different reward conditions [52]. This modified version uses non-monetary incentives (points) to invoke anticipation of reward and punishment. Participants’ outcome (score) is dependent upon their performance in a simple reaction time task, divided into control (no reward or punishment – 32 trials), potential reward (win 10 points – 32 trials) and potential punishment (lose 10 points – 32 trials) sub-tasks. The MIDT is sensitive to developmental and individual differences in sensitivity to cues for reward and punishment and three studies show relationships between ventral striatal activation on this task and risk taking and/or substance use in the IMAGEN sample [53–55]. The fMRI adaptation [56–59] of the Stop-Signal Task [60] measures activity in brain areas related to the inhibition of an already planned motor response as well as error detection. On a total of 300 trials, a motor response to high frequency go signals (75% of trials) has to be inhibited when infrequently and unexpectedly (in randomized 25% of trials), a stop signal appears after the go signal. The task is individually titrated to force every subject to fail on 50% of stop trials, making every subject work at the edge of their own inhibitory capacity, and therefore adjusting for differences in success levels between subjects and groups, making it ideal for developmental studies [56, 58, 59]. The fronto-striato-thalamic network during successful inhibition shows a progressive functional maturation between childhood and adulthood [56].

#### Sample size justification

An estimated 125 subjects in total will be required to identify a moderate effect of personality group (high SS, high IMP, low-risk) on brain measures, prior to the initiation of alcohol use, with 4 covariates, critical F(2, 118) = 3.07, *p* = 0.05, 85% power. Another 25 participants will be added to the sample to allow for attrition (15% over 4 years, based on previous studies). This sample size will provide 90% power to assess moderate group differences in developmental trajectories of alcohol use (group by time interactions) within a longitudinal design with 3 repeated assessments and corrections for multiple testing, critical F(4240) = 3.40, *p* = 0.001. The current study will be sufficiently powered to detect two-year changes on functional and structural data, considering numerous previous studies of similar size showing moderate (*r* = .30) age-related activation in cortical and subcortical regions of the brain when performing fMRI tasks tapping attention and higher cognitive abilities [10]. Similar power is required to test the effects of intervention.

#### Data analysis

Structural image analysis will be conducted with the cortical thickness and voxel based morphometry (VBM) analysis pipeline, CIVET (version 2.0) (http://www.bic.mni.mcgill.ca/ServicesSoftware/CIVET-2-0-0-Introduction. T1-weighted MRI images will be corrected for non-uniformity artifacts using the N3 algorithm, masked and registered into stereotaxic space, and then segmented into grey matter, white matter and cerebral spinal fluid using an advanced neural net classifier [61, 62]. The white and grey matter surfaces will be extracted using the Constrained Laplacian-based Automated Segmentation with Proximities algorithm [63]. The resulting surfaces will be resampled to a stereotaxic surface template to provide vertex based measures of cortical thickness. For each participant, cortical thickness will then be measured in native space using the linked distance between the two surfaces across 81,924 vertices and a 20 mm surface smoothing kernel will be applied to the data [64]. Statistical analyses will be performed using SurfStat (http://www.math.mcgill.ca/keith/surfstat/), a statistical toolbox created for MATLAB (The MathWorks, Inc., Nathan, MA, USA).

Diffusion images will be processed with FSL (FMRIB Diffusion Toolbox, http://www.fmrib.ox.ac.uk/fsl/). Motion artifacts and eddy current distortions will be corrected by using affine registration of DWI to the b0 image. Diffusion data will be registered to standard MNI152 space (2 mm isotropic) using a two-stage registration: rigid-body alignment and nonlinear transformation. A diffusion tensor model will be fitted for each voxel to estimate the principle fiber directions and calculate a fractional anisotropy (FA) map. Then, the probabilistic distribution of fiber orientations will be estimated using FSL’s BEDPOSTX with a maximum of 2 fiber directions per voxel [65]. The probabilistic tractography will be applied by sampling 10,000 streamline fibers per voxel within the seed region using FSL’s PROBTRACKX [66, 67].

Functional image analysis will be carried out using FSL (FEAT – FMRI Expert Analysis Tool). The following pre-processing steps will be applied: motion correction using MCFLIRT [68], non-brain removal using BET [69], and spatial smoothing with a Gaussian kernel of FWHM 6 mm. Time-series general linear model will be carried out using FILM with an autocorrelation correction [70]. At the first level, the following contrasts: anticipation/feedback of win and lose *vs* reference (MIDT) and successful and failed inhibition *vs* baseline (Stop-Signal Task) will be estimated for each participant. Group-level (second level) analysis will be carried out using a fixed effects model, by forcing the random effects variance to zero in FLAME (FMRIB’s Local Analysis of Mixed Effects) [71]. *Z* (Gaussianised T/F) statistic images will be thresholded using clusters determined by *Z* > 2.3 and a corrected cluster significance threshold of *p* = 0.05.

#### Statistical analyses

The main objectives of the statistical analyses will be to characterize differences for fMRI, cortical thickness, VBM, DWI and behavioral measures between high (SS and IMP) and low-risk youth, binge drinkers and non-drinker trajectory, and those assigned to the intervention or not (with and without exposure to alcohol) over time. Two- and three-way repeated measures analyses of covariance (ANCOVA) will be performed based on region of interest (ROI) scores with covariates such as age, socioeconomic status, pubertal stage, IQ, and other drug use. For cortical thickness and VBM only a-priori defined ROI will be used (i.e., prefrontal lobes, basal ganglia, limbic brain regions and corpus callosum), as previous studies have shown that alcohol affects these regions. For fMRI, we will conduct both exploratory and ROI analyses, and for the DWI data, the regions showing linear age trends in the fMRI analysis will be used as start/finish points for probabilistic tractography.

## Discussion

Imaging studies using different modalities have consistently reported that alcohol use during adolescence is associated with abnormalities in brain structure, function and connectivity [72]. However, the critical question of whether these anomalies are consequences of alcohol abuse or preexisting vulnerability factors for early use and misuse remains unanswered in the literature. This prospective, intervention study will allow us to identify brain, cognitive, and behavioral factors that precede (and predispose to) adolescent alcohol misuse versus factors that are consequential to such use. Moreover, this unique study will enable us to test whether an early alcohol prevention intervention produces protective effects on brain structure, function and connectivity in adolescents, while controlling for potential pre-morbid factors associated with a susceptibility to early onset drinking. By focusing on the neural correlates of adolescents at risk for harmful drinking, and by examining the effects of adolescent drinking and binge drinking on the development of brain structure and function, the study will extend the knowledge gained from other large-scale studies (e.g., European Commission FP6-Health www.imagen-europe.com and National Institutes of Health’s “Longitudinal Studies on the Impact of Adolescent Drinking on the Adolescent Brain”). Perhaps more importantly, this innovative experimental design will allow us to test the causal relationship between early alcohol exposure and neurocognitive and addiction outcomes by showing that delaying drinking in those most at-risk protects normal neurocognitive development.

The main strengths of the study are that (i) the selection, intervention and measurement models are highly time-sensitive, capturing critical periods (12–14 and 14–16 years of age) when personality endophenotypes translate to risk for binge drinking and when early intervention can produce preventative effects. Furthermore, (ii) this critical period appears to overlap with the age at which drinking onset is highly related to future drinking [73], and when important brain maturation occurs [10]. In addition, (iii) this research design is highly powered as our screening procedure, which is largely based on personality assessment at 12–13 years of age, identifies individuals, SS and IMP youth, for whom binge drinking is almost certain to develop within the next 12 months. Our previous research has shown that at 13 years of age, high SS and IMP adolescents have normal rates of drinking and binge drinking (around 40%), but experience a rapid rate of onset of binge drinking between 14 and 15 years of age, with approximately 95% reporting binge drinking at 15 years of age [34]. We will also be able to (iv) test critical questions on the causal pathway from premorbid risk markers to early onset drinking, disruptions in brain function and, in turn, addiction outcomes. Finally, by being embedded into a larger population-based 5-year longitudinal randomized controlled trial, we will be able to directly (v) test the generalizability of our sample and findings to the general population, which is unique for pediatric neuroimaging studies, which tend to involve highly selective clinical samples or families with high education and socio-economic characteristics.

Harmful alcohol use in early adolescence is associated with a more severe and protracted course of adult alcohol abuse and dependence and greater risk for severe psychiatric and other consequences of drinking. While these facts clearly indicate a need to focus our efforts on early intervention and prevention, particularly with vulnerable groups, only a fraction of the annual health budget dedicated to the treatment of substance abuse goes toward alcohol and drug prevention. This research will directly feed public knowledge on the harms associated with youth alcohol misuse, as well as the evidence base for selective interventions. The expected results will show direct effects of drinking on brain development and the benefits of early intervention and prevention efforts, effects that will be difficult to ignore in public policy on youth drinking.

## Abbreviations

BART: Balloon Analogue Risk Task
BOLD: Blood oxygenation level dependent
CIHR: Canadian Institutes of Health Research
CMS: Children’s memory scale
DWI: Diffusion weighted imaging
EPI: Echo planar imaging
FA: Fractional anisotropy
fMRI: Functional magnetic resonance imaging
IMP: Impulsivity
MIDT: Monetary Incentive Delay Task
MRI: Magnetic resonance imaging
ROI: Region of interest
SOPT: Self-Ordered Pointing Test
SS: Sensation-seeking
SURPS: Substance Use Risk Profile Scale
TE: Echo time
TR: Repetition time
VBM: Voxel based morphometry
